# Interferon-γ Receptor Signaling in Dendritic Cells Restrains Spontaneous Proliferation of CD4^+^ T Cells in Chronic Lymphopenic Mice

**DOI:** 10.3389/fimmu.2019.00140

**Published:** 2019-02-07

**Authors:** Laura Knop, Charlotte Frommer, Diana Stoycheva, Katrin Deiser, Ulrich Kalinke, Thomas Blankenstein, Thomas Kammertoens, Ildiko Rita Dunay, Thomas Schüler

**Affiliations:** ^1^Institute of Molecular and Clinical Immunology, Medical Faculty, Otto-von-Guericke University, Magdeburg, Germany; ^2^TWINCORE, Centre for Experimental and Clinical Infection Research, a joint venture between the Helmholtz Centre for Infection Research and the Medical School Hannover, Institute for Experimental Infection Research, Hannover, Germany; ^3^Institute of Immunology, Charité-Universitätsmedizin Berlin, Berlin, Germany; ^4^Max-Delbrück-Center for Molecular Medicine, Berlin, Germany; ^5^Berlin Institute of Health, Berlin, Germany; ^6^Institute of Inflammation and Neurodegeneration, Medical Faculty, Otto-von-Guericke University, Magdeburg, Germany

**Keywords:** CD4^+^ T cells, interferon-γ, lymphopenia, lymphopenia-induced proliferation (LIP), dendritic cells

## Abstract

In lymphopenic mice, T cells become activated and undergo lymphopenia-induced proliferation (LIP). However, not all T cells are equally sensitive to lymphopenia. Several lymphopenia-insensitive T cell clones were described and their non-responsiveness was mainly attributed to clone-specific properties. Here, we provide evidence for an additional, host-dependent mechanism restraining LIP of lymphopenia-insensitive CD4^+^ T cells. We show that such cells undergo LIP in lymphopenic mice lacking IFN-γ receptor (IFN-γR) expression, a process, which is promoted by the autocrine action of T cell-derived IFN-γ. Additionally, LIP of lymphopenia-insensitive CD4^+^ T cells requires an intact microflora and is accompanied by the massive accumulation of IL-6 and dendritic cells (DCs). Consistent with these results, IL-6 neutralization and the DC-specific restoration of IFN-γR expression are both sufficient to restrict LIP. Hence, the insensitivity of CD4^+^ T cells to lymphopenia relies on cell-intrinsic properties and a complex interplay between the commensal microflora, IL-6, IFN-γR^+^ DCs, and T cell-derived IFN-γ.

## Introduction

In lymphocyte-competent hosts, T cells continuously utilize homeostatic factors such as Interleukin-7 (IL-7) and self-peptide-MHC complexes and thereby limit their availability (1). Due to the lack of IL-7-consuming T cells, IL-7 accumulates in lymphopenic mice (2) and humans (3). IL-7 is a potent activation and survival signal for T cells and its overabundance promotes T cell responses (4). Consequently, the adoptive transfer of polyclonal naive CD4^+^ T cells into lymphopenic mice leads to their activation and subsequent lymphopenia-induced proliferation (LIP) (5, 6). However, LIP represents a mixed reaction in response to different stimuli. While IL-7 overabundance induces a comparably slow homeostatic proliferation (HP) of T cells, the commensal microflora triggers a rapid response referred to as spontaneous proliferation (SP) (7–11). Nevertheless, naive T cells undergoing LIP differentiate into interferon-γ (IFN-γ)-producing effector/memory T cells, which is frequently associated with autoimmunity (12, 13).

The degree of LIP varies strongly between T cell clones (14–16). For example, ovalbumin (OVA)-specific CD4^+^ TCR-transgenic (tg) OT-II T cells, contrary to polyclonal CD4^+^ T cells, do not undergo LIP in irradiated hosts (14) and expand only moderately in fully lymphopenic Rag-deficient (Rag^−/−^) mice (10). TCR signal strength is a major factor that regulates the sensitivity of a T cell to lymphopenia (15, 16). It is affected by a complex interplay between TCR avidity and molecules modulating TCR signal transduction (15, 17, 18). Hence, cell-intrinsic mechanisms appear to determine whether a T cell is sensitive to lymphopenia or not. However, it remained unclear whether extrinsic mechanisms prevent LIP of lymphopenia-insensitive CD4^+^ T cells.

In the present study, we show that lymphopenia-insensitive OT-II cells expand massively in IFN-γ receptor (IFN-γR)-deficient Rag^−/−^ (Rag^γ*Rko*^) mice, a phenomenon that is not observed in IFN-γ-deficient Rag^−/−^ (Rag^γ*ko*^) mice. LIP of OT-II cells is associated with a strong increase in systemic IL-6 and subsequent T cell accumulation. The lack of IFN-γ and IFN-γR expression by OT-II cells impaired LIP to some degree arguing for a growth promoting, autocrine effect of OT-II-derived IFN-γ. Furthermore, we show that the commensal microflora is crucial for OT-II LIP in Rag^γ*Rko*^ mice, which is accompanied by the massive expansion of dendritic cells (DCs). Finally, we show that IFN-γR expression exclusively in DCs is sufficient to restrict OT-II expansion, DC accumulation and IL-6 production in Rag^γ*Rko*^ mice. In summary, we provide evidence that the suppression of CD4^+^ T cell activation in response to lymphopenia is determined by a combination of both, clone-specific properties and environmental factors such as the commensal microflora, IL-6 and IFN-γR expression by DCs.

## Materials and Methods

### Mice and Adoptive T Cell Transfer

Thy1.1^+^ B6.PL-Thy1a/Cy and Thy1.2^+^ B6.129S7-Rag1^tm1Mom^/J (Rag^−/−^), C57BL/6J (B6), B6.SJL-Ptprc^a^Pepc^b^/BoyJ (CD45.1^+^), B6.129S7-Ifnγ^tm1Ts^ (IFN-γ^−/−^), B6.129S7-Ifngr^tm1Agt^ (IFN-γR^−/−^), B6.Cg-Tg(TcraTcrb)425Cbn/J (OT-II) (expressing a transgenic TCR specific for the chicken ovalbumin (OVA)-derived, I-A^b^-restricted peptide OVA_323−339_), B6.Cg-Tg(Itgax-EGFP-CRE-DTR-LUC)2Gjh/Crl (CD11c-GCDL) (19) and pCAG^loxP^STOP^loxP^-IFNγR-IRES-GFP (IFN-γR^SO^) transgenic mice (20) were housed under specific pathogen-free conditions. Mice were crossed to generate Thy1.1/.2/CD45.1/.2-disparate Rag^−/−^OT-II (OT-II^WT^), Rag^−/−^IFN-γR^−/−^OT-II (OT-II^γ*Rko*^), and Rag^−/−^IFN-γ^−/−^OT-II (OT-II^γ*ko*^) T cell donors. Lymphopenic Rag^−/−^ (Rag^WT^), Rag^−/−^IFN-γ^−/−^ (Rag^γ*ko*^), Rag^−/−^IFN-γR^−/−^ (Rag^γ*Rko*^), and Rag^−/−^IFN-γR^−/−^ × CD11c-GCDL × IFN-γR^SO^ (Rag^γ*Rko*^ × IFN-γR^CD11c−ON^) mice served as T cell recipients. For the adoptive transfers shown in Figures 2A,B, B6 or CD45.1^+^ mice served as non-lymphopenic controls. For T cell transfers, single cell suspensions were prepared from spleens and lymph nodes of donor mice by forcing the organs through metal sieves. To lyse erythrocytes, cell suspensions were incubated with Ammonium-Chloride-Potassium lysis buffer for 90 s and subsequent addition of RPMI with 10% FCS. After washing with PBS/2mM EDTA, cell suspensions were resuspended in PBS and filtered through 40 μm cell strainers (BD and Corning, Durham, NC). Single cell suspensions were counted, stained with fluorochrome-labeled antibodies for 30 min at 4°C and analyzed by flow cytometry to determine the frequency and activation state of OT-II cells (Supplementary Figure 1). Cell suspensions containing 1.6–10 × 10^5^ naive CD4^+^ OT-II T cells were injected i.v. into the tail vein of recipient mice. For CFSE labeling, donor single cell suspensions (2.2–3.2 × 10^7^ cells/ml) were incubated with 7.5 μM CFSE (Biolegend) in PBS for 20 min at 37°C. Subsequently, cells were washed twice with ice cold PBS or RPMI/10% FCS and were resuspended in PBS prior to injection. Cell suspensions containing 7.5–8 × 10^5^ CFSE^+^ OT-II T cells were injected i.v. into the tail vein of recipient mice. Ten to thirteen days after transfer, spleens and lymph nodes were isolated and single cell suspensions were prepared as described. Erythrocyte lysis was performed with spleen cell samples. Cells were counted and directly stained with fluorochrome-labeled antibodies for 30 min at 4°C after blocking FcR with purified anti-CD32/CD16 monoclonal antibodies (2.4G2 ATCC® HB-197™). To neutralize IL-6 *in vivo*, mice were i.p. injected with 500 μg of anti-IL-6 (MP5-20F3; BioXCell) 2 days prior to OT-II transfer. Treatment was repeated every third day. Control mice received 500 μg control IgG1 (HRPN; BioXCell). To deplete the commensal microflora, mice were treated with 0.5 g/l vancomycin, 1.0 g/l metronidazole, 1.0 g/l ampicillin, and 1.0 g/l neomycinsulfate via the drinking water 4 weeks prior to and during the experiment (21). Mice treated with antibiotics did not show any obvious clinical symptoms. At the day of analysis, however, their cecum was enlarged indicating successful depletion of the commensal microflora.

### Flow Cytometry

The following antibodies and reagents were used: anti-CD4 (RM4-5; Biolegend/eBioscience), -CD11c (N418; BD/Biolegend), -CD44 (IM7; Biolegend), -CD45.1 (A20; Biolegend), -CD62L (MEL-14; Biolegend), CD127 (A7R34; BD/Biolegend), -KLRG-1 (2F1; Biolegend/eBioscience), -Ki67 (SolA15; eBioscience), -I-A^b^ (AF6-120.1; Biolegend), -Thy1.1 (OX-7; Biolegend), -TCR Vα2 (B20.1; Biolegend), streptavidin-BV510 (Biolegend) and streptavidin-PE (Biolegend). For intranuclear staining of Ki67, cells were first stained with the indicated antibodies directed against cell surface molecules. Afterwards cells were fixed with the Foxp3/Transcription Factor Staining Buffer Set (eBioscience) according to the manufacturer's instructions and subsequently incubated with anti-Ki67 for 30 min at 4°C. Samples were measured on LSRFortessa flow cytometer (Becton Dickinson) and analyzed by FlowJo 9 and 10 software (FlowJo, LLC). To calculate the fold expansion of OT-II cells or DCs, the respective cell populations were quantified. For each experiment a mean value was calculated for the Rag^WT^ group. Finally, cell numbers of individual mice, including Rag^WT^ mice, were calculated in relation to the mean value of the Rag^WT^ group. Relative mean fluorescence intensities (MFIs) and relative frequencies of OT-II cells or DCs were calculated in analogy.

### IFN-γ and IL-6 Detection

Blood (supplemented with EDTA) was centrifuged 10 min at 500 × g and 4°C. The supernatant was centrifuged again 10 min at 900 × g and 4°C to obtain the plasma that was analyzed by an IFN-γ or IL-6 specific ELISA (eBioscience) according to manufacturer's instructions.

### Statistical Analysis

Statistical analysis and graphical representations were done using Prism 5 software (GraphPad Software). Statistical significance was determined using a non-parametric two-tailed Mann-Whitney *U*-test. ^*^p ≤ 0.05; ^**^p ≤ 0.01; ^***^p ≤ 0.001; ^****^p ≤ 0.0001.

## Results

### Host IFN-γR Expression Restrains Commensal-Driven OT-II LIP

**Figure 1:**
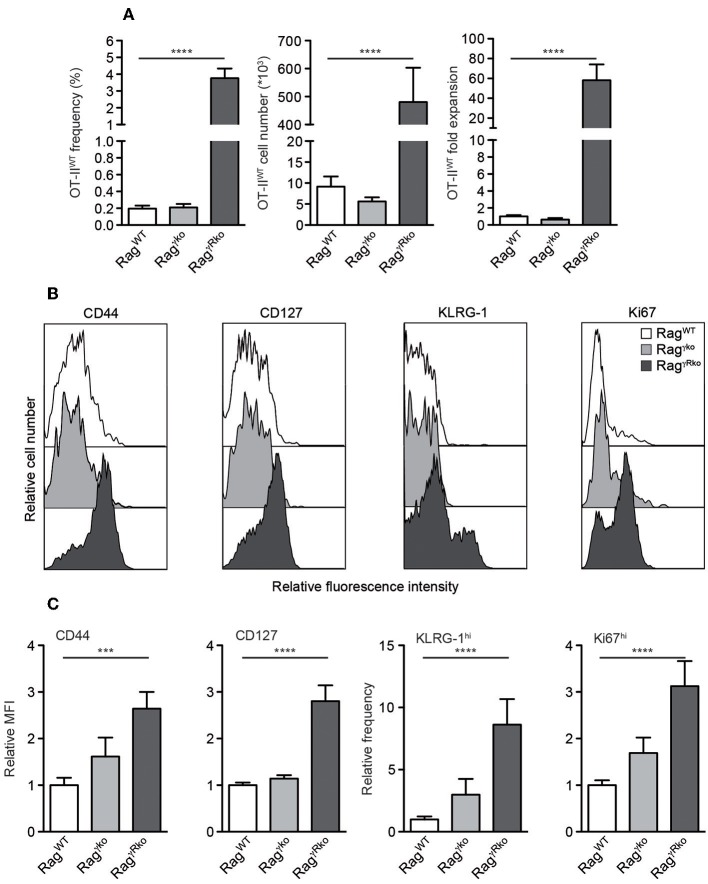
CD4^+^ T cell LIP is amplified in IFN-γR-deficient mice. **(A–C)** CD4^+^Thy1.1^+^ OT-II^WT^ T cells were adoptively transferred into Rag^WT^, Rag^γ*ko*^ and Rag^γ*Rko*^ mice (all Thy1.1^−^). After 10–12 days, recipient splenocytes were analyzed by flow cytometry. **(A)** Shown are frequencies, cell numbers and fold expansion of OT-II^WT^ cells. **(B)** Relative fluorescence intensities, **(C)** relative MFIs for CD44 and CD127 and relative frequencies of KLRG-1^hi^ and Ki67^hi^ cells were determined after gating on CD4^+^Thy1.1^+^ OT-II^WT^ cells. **(A,C)** Shown are pooled results from 3 to 4 independent experiments with a total of 11–17 mice per group and **(B)** representative histograms from corresponding samples. **(A,C)** Graphs show mean values + SEM and statistical significances (^***^p ≤ 0.001; ^****^p ≤ 0.0001) were calculated to values in Rag^WT^ mice.

**Figure 2:**
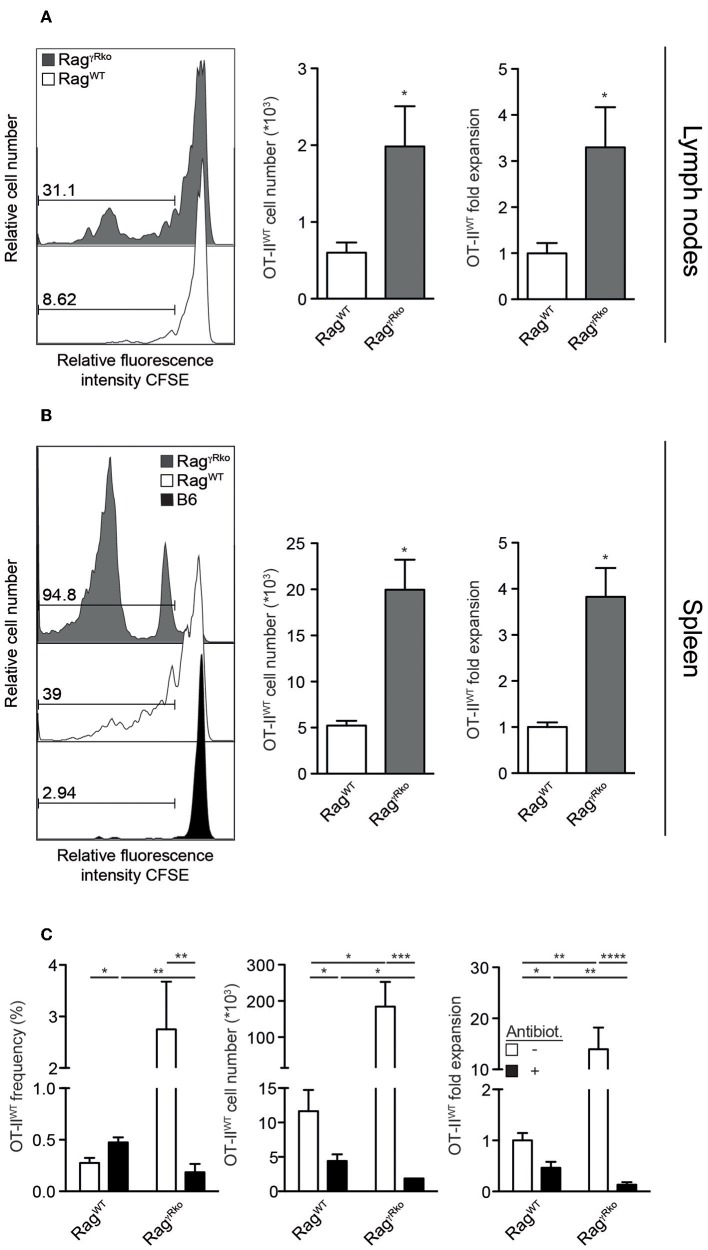
OT-II LIP is more pronounced in spleen than in lymph nodes. **(A,B)** CFSE-labeled OT-II^WT^ cells were adoptively transferred into Rag^WT^, Rag^γ*Rko*^ mice and **(B)** B6 mice. After 12 days, recipient **(A)** lymph nodes and **(B)** spleen were analyzed by flow cytometry. **(A,B)** Histograms show relative fluorescence intensities for CFSE after gating on CD4^+^CD45.1^+^ OT-II^WT^ cells and numbers indicate percentages. Bar diagrams show cell numbers and fold expansion of OT-II^WT^ cells (mean values + SEM; ^*^p ≤ 0.05). Results in bar diagrams were pooled from 6 mice per group analyzed in one experiment. **(A)** Histograms are representative of one experiment with 6 Rag^WT^ and 6 Rag^γ*Rko*^. **(B)** Histograms are representative of 2 independent experiments with a total of 10 Rag^WT^, 10 Rag^γ*Rko*^, and 4 B6 mice. **(C)** OT-II^WT^ cells were adoptively transferred into Rag^WT^ and Rag^γ*Rko*^ mice. After 11–13 days, recipient splenocytes were analyzed by flow cytometry. Four weeks prior to and during T cell transfer, mice were treated with antibiotics (Antibiot.) or were left untreated. Shown are pooled results (mean values + SEM; ^*^p ≤ 0.05; ^**^p ≤ 0.01; ^***^p ≤ 0.001; ^****^p ≤ 0.0001) from 2 independent experiments with a total of 8–9 mice per group.

We have shown that host IFN-γR signaling restricts LIP of CD8^+^ T cells (22). Whether this mechanism prevents LIP of CD4^+^ OT-II T cells was unclear. To address this issue, naive CD4^+^ T cells from Rag^−/−^ OT-II TCR^tg^ mice (OT-II^WT^ cells) were adoptively transferred into IFN-γR-deficient Rag^−/−^ (Rag^γ*Rko*^) and IFN-γR-competent Rag^−/−^ (Rag^WT^) mice. To elucidate a potential contribution of host-derived IFN-γ, IFN-γ-deficient Rag^−/−^ mice (Rag^γ*ko*^) were reconstituted with OT-II^WT^ cells in parallel. Within 10–12 days, OT-II^WT^ cells expanded massively in Rag^γ*Rko*^ but not in Rag^WT^ or Rag^γ*ko*^ spleens (Figure 1A). LIP was associated with the up-regulation of CD44, CD127, KLRG-1, and Ki67 indicating full activation and proliferation of OT-II^WT^ cells in Rag^γ*Rko*^ mice (Figures 1B,C). LIP is induced in T cell areas of secondary lymphoid organs (SLOs) (23) and IFN-γ regulates T cell migration to and positioning in SLOs (24–26), which is guided by chemokine-producing stromal cells (27). However, stromal cell composition differs significantly between lymph nodes (LNs) and spleen (28). We therefore asked next whether OT-II expansion is equally well induced in either SLO. To address this question, CFSE-labeled OT-II^WT^ cells were transferred into Rag^WT^ and Rag^γ*Rko*^ mice. C57BL/6 (B6) served as non-lymphopenic controls. After 12 days, recipient LNs and spleens were analyzed. As shown in Figures 2A,B, the frequencies of CFSE^lo^ OT-II^WT^ cells were lower in LNs than in spleen of both recipients. However, CFSE^lo^ OT-II^WT^ cells were clearly more abundant in Rag^γ*Rko*^ spleens and LNs (Figures 2A,B) indicating higher frequencies of rapidly dividing OT-II^WT^ cells in either organ. Of note, in addition to the rapidly dividing CFSE^lo^ OT-II cells, a population of CFSE^int^ cells was detectable in the spleen, but not LNs, of Rag^γ*Rko*^ mice (Figures 2A,B). This suggests different, organ-specific velocities of OT-II LIP. Nonetheless, OT-II^WT^ LIP was most pronounced in the spleens of Rag^γ*Rko*^ mice. We therefore focused on this organ in the following experiments.

Under lymphopenic conditions, the rapid-type of T cell proliferation relies on the presence of an intact commensal microflora (7, 10). Whether this is also the case for OT-II expansion in Rag^γ*Rko*^ mice was studied next. For this purpose, Rag^WT^ and Rag^γ*Rko*^ mice were treated with a mixture of antibiotics prior to and during reconstitution with OT-II^WT^ cells. This treatment regimen efficiently depletes commensals (21, 29). As expected, OT-II^WT^ expansion was impaired in untreated Rag^WT^ mice but was very efficient in untreated Rag^γ*Rko*^ mice (Figure 2C, white bars). On the contrary, antibiotic treatment blocked OT-II^WT^ LIP in Rag^γ*Rko*^ mice (Figure 2C). Together, the data presented so far indicate that recipient IFN-γR expression restrains commensal-driven spontaneous proliferation (SP) (7–11) of OT-II cells under lymphopenic conditions.

### IL-6 Accumulates in Rag^γ*Rko*^ Mice and Promotes OT-II SP

**Figure 3:**
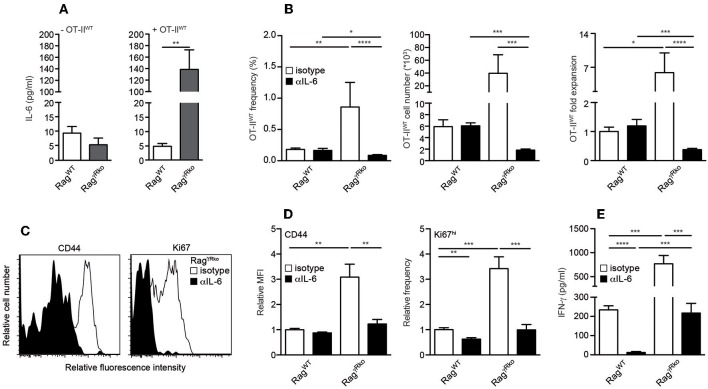
IL-6 accumulates in Rag^γ*Rko*^ mice and promotes OT-II SP. **(A–E)** Rag^WT^ and Rag^γ*Rko*^ mice were reconstituted with OT-II^WT^ cells as described in Figure 1. **(A)** Untreated mice served as controls (-OT-II^WT^). **(B–D)** Prior to and after T cell reconstitution, mice were treated with neutralizing anti-IL-6 (αIL-6) or isotype-machted control antibodies (isotype). Ten to twelve days after T cell transfer, **(A)** IL-6 and **(E)** IFN-γ plasma levels were determined by ELISA and **(B–D)** recipient splenocytes were analyzed by flow cytometry. **(B)** Shown are frequencies, cell numbers and fold expansion of OT-II^WT^ cells in isotype- and αIL-6-treated Rag^WT^ and Rag^γ*Rko*^ mice. **(C)** Relative fluorescence intensities, **(D)** relative MFIs for CD44 and relative frequencies of Ki67^hi^ cells were determined after gating on CD4^+^Thy1.1^+^ OT-II^WT^ cells in isotype- and αIL-6-treated Rag^γ*Rko*^ mice. **(A,B,D,E)** Shown are pooled results from 2 to 3 independent experiments with a total of 5–11 mice per group and **(C)** representative histograms from corresponding samples. **(A,B,D,E)** Graphs show mean values + SEM; ^*^p ≤ 0.05; ^**^p ≤ 0.01; ^***^p ≤ 0.001; ^****^p ≤ 0.0001.

IL-6 promotes commensal-dependent SP of CD4^+^ and CD8^+^ T cells in lymphopenic mice (9, 10). To elucidate whether IL-6 levels are altered in our experimental system, plasma samples from OT-II^WT^-reconstituted Rag^WT^ and Rag^γ*Rko*^ were analyzed 10–12 days after T cell transfer. As shown in Figure 3A, plasma levels of IL-6 were strongly elevated in OT-II^WT^-reconstituted Rag^γ*Rko*^ mice (Figure 3A; + OT-II^WT^) but not in untreated controls (Figure 3A; –OT-II^WT^). In order to test whether IL-6 promotes OT-II^WT^ SP in Rag^γ*Rko*^ mice, Rag^WT^, and Rag^γ*Rko*^ mice were treated with neutralizing monoclonal anti-IL-6 antibodies (αIL-6 mAb) prior to and after reconstitution with OT-II^WT^ cells. Control mice received isotype-matched control mAbs. As shown in Figure 3B, αIL-6 treatment did not affect frequencies, cell numbers or relative expansion rates of OT-II^WT^ cells in Rag^WT^ mice. As expected, OT-II^WT^ cells were by far most abundant in isotype-treated Rag^γ*Rko*^ mice, an effect that was fully reverted by IL-6 neutralization. Accordingly, expression levels of CD44 and Ki67 were strongly reduced in OT-II^WT^ cells recovered from αIL-6-treated Rag^γ*Rko*^ mice as compared to isotype-treated controls (Figures 3C,D). Hence, IL-6 is up-regulated upon T cell transfer and is crucial for OT-II^WT^ activation, proliferation and subsequent accumulation in Rag^γ*Rko*^ mice.

### OT-II-Derived IFN-γ Promotes SP in an Autocrine Fashion

T cell-intrinsic IL-6R signaling promotes the expansion of IFN-γ-producing effector/memory CD4^+^ T cells under lymphopenic and non-lymphopenic conditions (30, 31). Consequently, the blockade of OT-II^WT^ activation and subsequent SP in αIL-6-treated Rag^γ*Rko*^ mice (Figures 3B–D) correlated with a strong reduction of plasma IFN-γ levels (Figure 3E).

**Figure 4:**
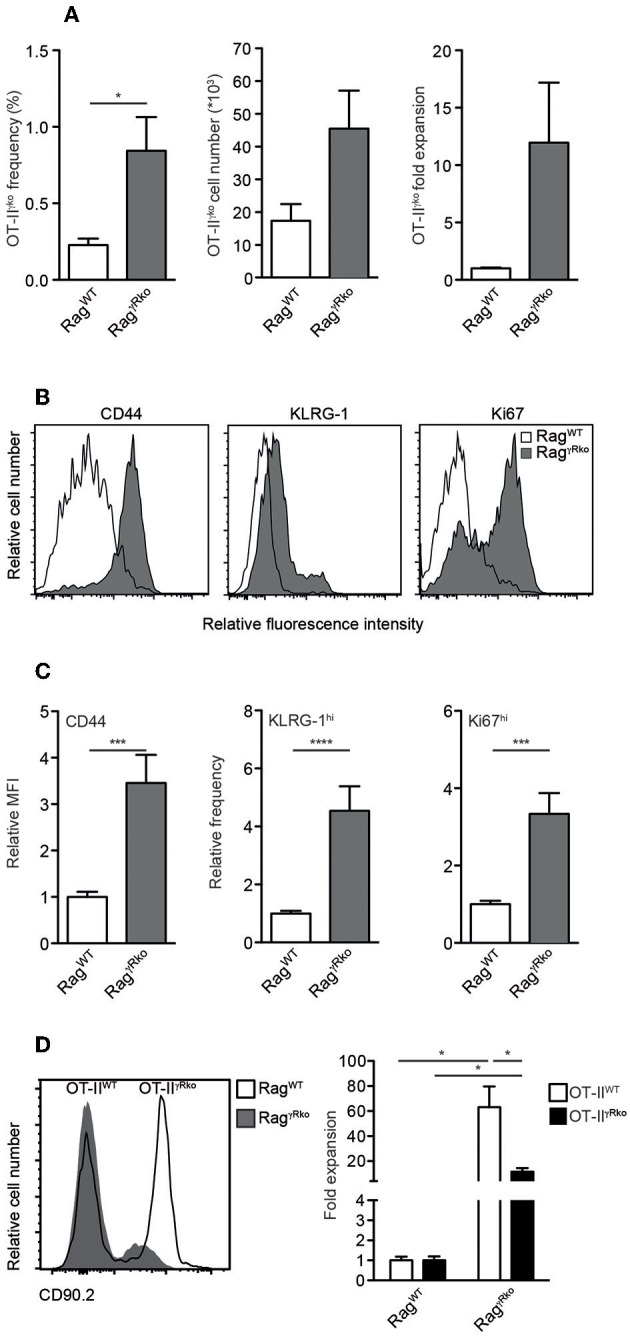
CD4^+^ T cell-derived IFN-γ promotes SP in an autocrine manner. **(A–C)** OT-II^γ*ko*^ (3–4 independent experiments with 12–17 mice per group) or **(D)** equal numbers of Thy1.1^+^ OT-II^WT^ and Thy1.1/1.2^+^ OT-II^γ*Rko*^ T cells (4 mice per group) were transferred simultaneously into Thy1.2^+^ Rag^WT^ and Rag^γ*Rko*^ mice. After 11–12 days, recipient splenocytes were analyzed by flow cytometry as described in Figure 1. Overlay shows the relative abundance of Thy1.1^+^ OT-II^WT^ and Thy1.1/1.2^+^ OT-II^γ*Rko*^ T cells in Rag^WT^ and Rag^γ*Rko*^ mice. **(A,C,D)** Graphs show mean values + SEM; ^*^p ≤ 0.05; ^***^p ≤ 0.001; ^****^p ≤ 0.0001.

Since IFN-γ directly promotes CD4^+^ T cell responses (32–34), we hypothesized that OT-II-derived IFN-γ supports SP in Rag^γ*Rko*^ mice in an autocrine fashion. To test this hypothesis, IFN-γ-deficient OT-II (OT-II^γ*ko*^) cells were transferred into Rag^γ*Rko*^ and Rag^WT^ mice. After 11–12 days, OT-II^γ*ko*^ frequencies, cell numbers and relative expansion rates were determined. As shown in Figure 4A, some expansion of OT-II^γ*ko*^ cells was detectable in Rag^γ*Rko*^. This was associated with the up-regulation of CD44, KLRG-1 and Ki67 (Figures 4B,C). Importantly, however, OT-II^γ*ko*^ cells expanded less well in Rag^γ*Rko*^ mice (~10-fold; Figure 4A) than OT-II^WT^ cells (~50-fold; Figure 1A) suggesting a growth-promoting effect of autocrine IFN-γ.

To further test this possibility, equal numbers of OT-II^WT^ and OT-II^γ*Rko*^ cells were co-transferred into Rag^γ*Rko*^ and Rag^WT^ mice. OT-II^WT^ cells expanded ~60-fold while OT-II^γ*Rko*^ cells expanded only ~20-fold (Figure 4D). Thus, SP of OT-II^γ*ko*^ and OT-II^γ*Rko*^ cells occurs in Rag^γ*Rko*^ mice. Compared to OT-II^WT^ cells, OT-II^γ*ko*^ and OT-II^γ*Rko*^ expansion was less pronounced suggesting that OT-II-derived IFN-γ promotes SP in an autocrine fashion. However, we cannot exclude a contribution of host-derived IFN-γ, which accumulates in IFN-γR-deficient mice due to lack of its consumption (22).

### IFN-γR^+^ DCs Restrain CD4^+^ T Cell SP in Rag^γ*Rko*^ Mice

**Figure 5:**
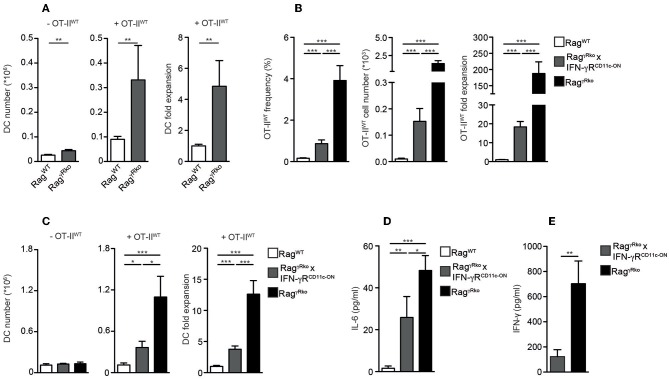
IFN-γR^+^ DCs restrain CD4^+^ T cell SP in Rag^γ*Rko*^ mice. **(A–D)** OT-II^WT^ cells were adoptively transferred into Rag^WT^ and Rag^γ*Rko*^ mice. After 11–13 days, recipient splenocytes were analyzed by flow cytometry. **(A)** Results of 2–6 independent experiments with a total of 10–25 mice were pooled to calculate the numbers and fold expansion of CD11c^+^MHCII^hi^ DCs after reconstitution with OT-II^WT^ cells (+OT-II^WT^). DC numbers from untreated Rag^WT^ and Rag^γ*Rko*^ mice were determined as well (-OT-II^WT^). **(B–D)** Frequencies, cell numbers and fold expansion of OT-II^WT^ cells/DCs as well as plasma IL-6 levels were analyzed in Rag^WT^, Rag^γ*Rko*^ × CD11c-GCDL × IFN-γR^SO^ (Rag^γ*Rko*^ × IFN-γR^CD11c−ON^) and Rag^γ*Rko*^ mice. Pooled results of 2 independent experiments with a total of 8 mice per group are shown. **(E)** Steady-state levels of IFN-γ were determined in plasma samples of 8–9 untreated Rag^γ*Rko*^ × IFN-γR^CD11c−ON^ and Rag^γ*Rko*^ mice. **(A–E)** Graphs show mean values + SEM; ^*^p ≤ 0.05; ^**^p ≤ 0.01; ^***^p ≤ 0.001.

Dendritic cells (DCs) producing elevated levels of IL-6 promote aberrant T cell activation and subsequent IFN-γ synthesis (35). Furthermore, the induction of EAE relies on the accumulation of IL-6-producing DCs (36). Under lymphopenic conditions, MyD88-dependent recognition of the commensal microflora is sufficient to induce IL-6 production by DCs thereby promoting SP of CD4^+^ T cells (10) similar to what we have observed in OT-II^WT^-reconstituted Rag^γ*Rko*^ mice. Furthermore, DCs express high levels of MHCII, which is crucial for CD4^+^ T cell LIP (14, 37). Based on these data we speculated that DC responses were altered in Rag^γ*Rko*^ mice. When splenic CD11c^+^MHCII^hi^ DCs were quantified in OT-II^WT^-reconstituted Rag^WT^ and Rag^γ*Rko*^ mice, their numbers were strongly increased in the latter (Figure 5A; + OT-II^WT^). This was not the case in untreated Rag^γ*Rko*^ mice (Figure 5A; –OT-II^WT^) suggesting that OT-II^WT^ activation is a prerequisite for DC accumulation in Rag^γ*Rko*^ recipients.

Whether the DC-specific restoration of IFN-γR expression is sufficient to block OT-II^WT^ SP and subsequent DC accumulation in Rag^γ*Rko*^ mice was tested next. For this purpose, we made use of a novel transgenic mouse line, allowing IFN-γR expression after the Cre-mediated deletion of a loxP-flanked DNA-Stop cassette (20). To activate this “switch-on” (IFN-γR^SO^) construct and express the transgenic IFN-γR specifically in DCs, IFN-γR^SO^ mice were crossed to CD11c-GCDL mice expressing Cre under the control of the CD11c promoter (19). Subsequently, CD11c-GCDL × IFN-γR^SO^ mice were crossed to Rag^γ*Rko*^ mice in order to generate T and B cell-deficient, fully lymphopenic Rag^γ*Rko*^ × CD11c-GCDL × IFN-γR^SO^ mice lacking IFN-γR expression on all cells except DCs. These mice are termed Rag^γ*Rko*^ × IFN-γR^CD11c−ON^ hereafter. Finally, OT-II^WT^ cells were transferred into Rag^WT^ mice, Rag^γ*Rko*^ × IFN-γR^CD11c−ON^, and Rag^γ*Rko*^ controls. After 11–13 days, the numbers of splenic OT-II^WT^ cells were determined. As opposed to Rag^WT^ mice, OT-II^WT^ cells expanded strongly in Rag^γ*Rko*^ mice (Figure 5B). The values obtained with Rag^γ*Rko*^ × IFN-γR^CD11c−ON^ mice reached intermediate levels showing that IFN-γR expression by DCs is sufficient to restrain OT-II^WT^ SP. Similarly, DC expansion was most pronounced in OT-II^WT^-reconstituted Rag^γ*Rko*^ mice, reached intermediate levels in Rag^γ*Rko*^ × IFN-γR^CD11c−ON^ mice and was least efficient in Rag^WT^ mice (Figure 5C; +OT-II^WT^). On the contrary, DC numbers did not differ between untreated Rag^WT^, Rag^γ*Rko*^ × IFN-γR^CD11c−ON^ and Rag^γ*Rko*^ mice (Figure 5C; –OT-II^WT^) suggesting a causal link between OT-II^WT^ SP and DC expansion in Rag^γ*Rko*^ mice (Figures 5A,C). Importantly, specific IFN-γR expression by DCs was sufficient to limit expansion of OT-II^WT^ cells and DCs as well as IL-6 up-regulation (Figure 5D) in Rag^γ*Rko*^ × IFN-γR^CD11c−ON^ mice.

The efficacy of CD4^+^ T cell responses correlates positively with the amount of IFN-γ available in the early phase of the response (32, 34). We have shown previously that IFN-γ accumulates in IFN-γR-deficient mice, most probably due to the lack of its receptor-mediated clearance (22). Hence, elevated levels of steady-state IFN-γ may explain the rapid and strong induction of OT-II^WT^ responses in Rag^γ*Rko*^ mice. To test whether decreased OT-II^WT^ responses in Rag^γ*Rko*^ × IFN-γR^CD11c−ON^ mice (Figure 5B) correlate with reduced steady-state IFN-γ levels, we compared plasma samples of untreated Rag^γ*Rko*^ and Rag^γ*Rko*^ × IFN-γR^CD11c−ON^ mice. As shown in Figure 5E, IFN-γ levels were significantly lower in Rag^γ*Rko*^ × IFN-γR^CD11c−ON^ mice. This suggests that IFN-γR^+^ DCs consume IFN-γ thereby reducing its availability for OT-II^WT^ cells. This competition for IFN-γ would provide an explanation for the reduced levels of SP in Rag^γ*Rko*^ × IFN-γR^CD11c−ON^ mice (Figure 5B).

## Discussion

T cell clones are not equally sensitive to lymphopenia-related activation signals (14–16). For example, ovalbumin-specific CD4^+^ T cells from OT-II TCR^tg^ mice represent one of several T cells clones, which are resistant to lymphopenia-induced activation (14). It is well accepted that T cell clone-specific features such as CD5 levels correlate closely with the sensitivity to lymphopenia (15, 16, 38). Here, we provide evidence for an additional, recipient-dependent mechanism that restrains expansion of adoptively transferred CD4^+^ T cells. This mechanism relies on a complex interplay between the commensal microflora, IFN-γR^+^ DCs and CD4^+^ T cells.

The commensal microflora triggers IFN-γ production by various immune cells in the steady-state (39, 40). In IFN-γR-deficient mice, IFN-γ accumulates due to the lack of its consumption (22). Thus, elevated IFN-γ levels in Rag^γ*Rko*^ mice may provide early activation signals to OT-II cells initiating the rapid expansion we have observed. This interpretation is in accordance with our finding that both, OT-II^WT^ expansion and steady-state levels of IFN-γ, were decreased in Rag^γ*Rko*^ × IFN-γR^CD11c−ON^ mice. This suggests that IFN-γR^+^ DCs efficiently reduce amounts of circulating IFN-γ thereby restricting its availability for OT-II cells.

However, increased rates of OT-II expansion in Rag^γ*Rko*^ mice do not only rely on host-derived IFN-γ. As we have shown here, OT-II-derived IFN-γ acts in an autocrine manner. Hence, host- and OT-II-derived IFN-γ may synergize in promoting full-blown OT-II expansion in Rag^γ*Rko*^ mice. OT-II expansion is accompanied by the up-regulation of CD127, which would facilitate their IL-7-dependent survival (41–43) and provides one explanation for the accumulation of OT-II cells in Rag^γ*Rko*^ mice. Importantly, the accumulation of DCs and IL-6 correlates positively with the degree of OT-II expansion in Rag^γ*Rko*^ mice and might be interrelated. DCs produce IL-6 in response to the commensal microflora (10) and express MHCII, which are both required for CD4^+^ T cell expansion under lymphopenic conditions (10, 14, 37). Since (i) T cell-intrinsic IL-6R signaling is critical for CD4^+^ T cell responses (30, 31), (ii) IL-6 prevents apoptosis of naive and effector CD4^+^ T cells (44, 45), and (iii) counter-regulates DC function (35, 46–50) we suggest a direct, growth-promoting and/or anti-apoptotic effect of IL-6 on OT-II cells expanding in Rag^γ*Rko*^ mice. Although the T cell-stimulatory potential of DC-derived IL-6 is well established (10, 35, 36) recent findings identified multiple hematopoietic and non-hematopoietic cell types as potential IL-6 producers (36). Importantly, different IL-6 producers appear to regulate different aspects of the same CD4^+^ T cell response (36). Hence, it remains to be shown for our experimental system whether (i) DCs and/or other cell types up-regulate IL-6 expression in OT-II-reconstituted Rag^γ*Rko*^ mice, whether (ii) the elevation of IL-6 levels in these mice results from the accumulation of DCs producing constant amounts of IL-6, and whether (iii) there is a causal relationship between the cellular origin of IL-6 and its growth-promoting effect. As reported only recently, definite answers to such questions would require the combined use of cell type-specific IL-6 reporter as well as conditional IL-6 knockout mice (36) and their integration into our experimental systems. However, this would be beyond the scope of this study and therefore remains an important task for the future.

From previous experiments we know that only effector, but not naive, OT-II^WT^ cells activate immature DCs (51). This suggests that IFN-γ-associated OT-II activation is an integral part of a self-amplifying loop in Rag^γ*Rko*^ mice, which involves the T cell-dependent accumulation of DCs, which in turn promote OT-II expansion. The lack of IFN-γR signaling in DCs increases their lifespan (52) and T cell-stimulatory potential (53) providing an additional explanation for the accumulation of DCs in Rag^γ*Rko*^ mice. In accordance with this interpretation, IFN-γR re-expression in DCs is sufficient to disrupt this self-amplifying loop and to down-modulate DC accumulation, IL-6 levels and OT-II cell expansion.

In summary, we demonstrate that the sensitivity of CD4^+^ T cells to lymphopenia is not only determined by cell-intrinsic properties but also by a complex interplay between CD4^+^ T cells, the commensal microflora and IFN-γR^+^ DCs. We postulate that T cell- and host cell-specific mechanisms have to co-operate to restrain spontaneous proliferation, the commensal-driven form of LIP. The molecular nature and the relative importance of either mechanism may vary for different T cell clones.

## Ethics Statement

Animal experiments were performed according to institutional guidelines and were approved by the Landesverwaltungsamt Sachsen-Anhalt (Permit Number: 2-1155/2-1288 Uni MD).

## Author Contributions

LK, CF, DS, and KD performed and analyzed the experiments. LK substantially contributed to manuscript preparation. UK and ID analyzed and discussed the data. TB and TK provided essential material, analyzed and discussed the data. TS designed and supervised the study, analyzed and discussed the data and wrote the manuscript with the help of the other co-authors.

### Conflict of Interest Statement

The authors declare that the research was conducted in the absence of any commercial or financial relationships that could be construed as a potential conflict of interest.
