# Supplementary material for: Interferon-γ Receptor Signaling in Dendritic Cells Restrains Spontaneous Proliferation of CD4+ T Cells in Chronic Lymphopenic Mice
Source: Front Immunol. 2019 Feb 7;10:140. doi: 10.3389/fimmu.2019.00140 (PMC6374634; doi:10.3389/fimmu.2019.00140)
Supplement: Supplementary file 1 [file Data_Sheet_1.PDF]

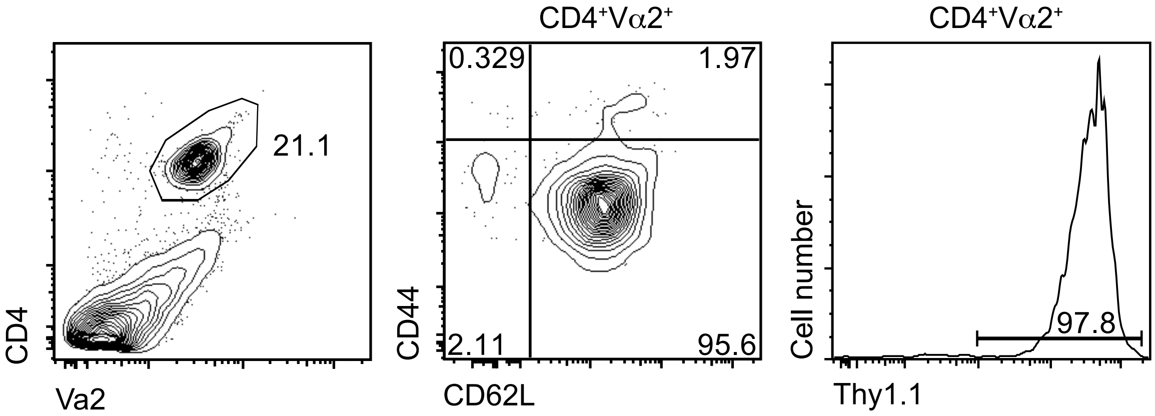

Supplementary Figure 1

Purity and phenotype of transferred OT-II^{WT} T cells. Single cell suspensions prepared from spleens and lymph nodes of Rag$^{-/-}$OT-II (OT-II^{WT}) mice were analyzed by flow cytometry to determine OT-II cell frequency and activation state prior to adoptive transfer. Shown are representative results. Numbers indicate percentages.
